# Reducing stigma and promoting HIV wellness/mental health of sexual and gender minorities: RCT results from a group‐based programme in Nigeria

**DOI:** 10.1002/jia2.26256

**Published:** 2024-06-05

**Authors:** Julie Pulerwitz, Ann Gottert, Waimar Tun, Anita Fernandez Eromhonsele, Progress Lanre Oladimeji, Elizabeth Shoyemi, Mauton Akoro, Columbus Ndeloa, Adebola Adedimeji

**Affiliations:** ^1^ Social and Behavioural Science Research Division Population Council Washington DC USA; ^2^ International Programs, Population Council Abuja Nigeria; ^3^ CPHI Lagos Nigeria; ^4^ TIERS Lagos Nigeria; ^5^ Elton John AIDS Foundation London UK

**Keywords:** Africa, cognitive behavioural therapy, HIV, mental health, MSM, PrEP, stigma, transgender

## Abstract

**Introduction:**

High levels of HIV stigma as well as stigma directed towards sexual and/or gender minorities (SGMs) are well documented in the African setting. These intersecting stigmas impede psychosocial wellbeing and HIV prevention and care. Yet, there are few if any evidence‐based interventions that focus on reducing internalized stigma and promoting mental health and HIV wellness for SGMs in Africa. We developed and evaluated a group‐based intervention drawing on cognitive behavioural therapy (CBT) strategies for men who have sex with men (MSM) and transgender women (TGW) at risk for or living with HIV in Lagos, Nigeria.

**Methods:**

The intervention comprised four weekly in‐person group sessions facilitated by community health workers. We conducted a delayed intervention group randomized controlled trial (April−September 2022), with pre‐post surveys plus 3‐month follow‐up (immediate group only), as well as qualitative research with participants and programme staff. Outcomes included internalized stigma related to SGM and HIV status, depression, resiliency/coping and pre‐exposure prophylaxis (PrEP)/HIV treatment use.

**Results:**

Mean age of the 240 participants was 26 years (range 18−42). Seventy‐seven percent self‐identified as MSM and 23% TGW; 27% were people with HIV. Most (88%) participants attended all four sessions, and 98% expressed high intervention satisfaction. There was significant pre‐post improvement in each psychosocial outcome, in both the immediate and delayed arms. There were further positive changes for the immediate intervention group by 3‐month follow‐up (e.g. in intersectional internalized stigma, depression). While baseline levels of ever‐PrEP use were the same, 75% of immediate‐group participants reported currently using PrEP at 3 months post‐intervention versus 53% of delayed‐group participants right after the intervention (*p*<0.01). Participants post‐intervention described (in qualitative interviews) less self‐blame, and enhanced social support and resilience when facing stigma, as well as motivation to use PrEP, and indicated that positive pre‐intervention changes in psychosocial factors found in the delayed group mainly reflected perceived support from the study interviewers.

**Conclusions:**

This study demonstrated the feasibility and acceptability of a group‐based CBT model for MSM and TGW in Nigeria. There were also some indications of positive shifts related to stigma, mental health and PrEP, despite issues with maintaining the randomized design in this challenging environment.

## 1 INTRODUCTION

High levels of HIV stigma as well as stigma directed towards sexual and gender minorities (SGMs) are well documented globally, and increasingly in sub‐Saharan Africa (SSA) [1, 2, 3, 4, 5, 6, 7, 8]. The experience of anti‐SGM and HIV stigma—as well as other stigmas such as those related to ethnicity or socio‐economic status—can “intersect,” leading to compounding negative effects on mental and physical health [9, 10, 11, 12, 13, 14]. Stigmatizing views that are internalized (also called self‐stigma) have been directly linked to poor mental health and inhibit the uptake of HIV services like pre‐exposure prophylaxis (PrEP) and antiretroviral therapy (ART) [1, 14–16].

SGMs in SSA experience a high burden of HIV and sexually transmitted infections [17, 18, 19, 20], physical and sexual violence [21, 22], mental health issues [7, 22] and inadequate access to HIV prevention services [13]. According to the Nigerian Federal Ministry of Health, the estimated HIV prevalence among men who have sex with men (MSM) in the country is 25% and among transgender persons is 29% [23], with many not being aware of their status [24]. SGMs also often have to navigate various forms of stigma, including laws criminalizing same‐sex relationships [25, 26]. Nigeria's Same Sex Marriage Prohibition Act (2013) is an example of such a law [27].

In recent years, several anit‐SGM stigma reduction efforts (primarily in the context of HIV service promotion) have been documented in SSA countries [28], including health provider training and community‐based campaigns to shift stigmatizing attitudes [29, 30, 31]. However, there are few evidence‐based interventions in the African context (or in fact, globally) designed specifically to address internalized stigma and resulting mental health and HIV self‐care challenges deriving from the multiple stigmas that SGMs face.

Developing and testing innovative interventions are clearly needed to reduce internalized stigma and its negative effects, and to facilitate resilience (or positive responses in the face of adversity). Cognitive behavioural therapy (CBT) is a promising approach for doing so. CBT is among the most impactful approaches for supporting mental health and reducing psychosocial distress [32, 33, 34]. In recent years, it has become more common for CBT to be implemented in a small group setting rather than individually, to foster social support [35, 36, 37]. Group‐based CBT can provide opportunities for a safe space to discuss challenges and develop shared responses to the challenges faced, enhance connection to and support from peers and other allies, and make links to health and wellness options/services [38].

This paper describes the development and evaluation of a group‐based internalized stigma reduction and resiliency‐promoting intervention for MSM and transgender women (TGW) in Lagos, Nigeria. The programme drew upon CBT strategies, and applied an intersectional lens to understanding and responding to stigma. We sought to evaluate intervention acceptability and feasibility, as well as preliminary efficacy to improve participants’ psychosocial wellbeing and use of HIV services.

## 2 METHODS

We conducted a delayed intervention randomised controlled trial, which included pre‐post surveys plus a 3‐month follow‐up (immediate group only), as well as in‐depth interviews (IDIs) and focus group discussions (FGDs) with participants and programme staff, from April to September 2022. Ethical approval for the study was given by the Population Council Institutional Review Board and the Nigerian Institute of Medical Research, and the study is registered through the ISRCTN Registry (ISRCTN95309635). All study participants completed written informed consent for every study activity.

### 2.1 Intervention

The intervention consisted of four weekly in‐person sessions 2.5–3 hours in length each, which were facilitated by Community Health Education Workers (CHEWs) with basic counselling skills, and with experience working with SGM populations in Lagos. Ten CHEWs completed a five full‐day interactive training by a nurse counsellor with CBT experience. Five CHEWs were selected to be facilitators. Sessions took place in community‐based organizations that employed the CHEWs and offered HIV and other health services to the SGM community. There were 10−12 self‐identified MSM or TGW participants per group. Given the focus on mental health and HIV self‐care—regardless of HIV status—for the SGM community (and feedback from local service providers), this was developed as a “status‐neutral” intervention. Groups were not separated by HIV status, and curriculum content related to HIV and HIV‐related stigma was carefully designed to be applicable to and generate reflection among all participants. To ensure quality, one programme manager was present at every session and participants completed a feedback form at the end of each session. Participants were informed from the start that they could access a counsellor/psychologist free of charge during their participation.

The intervention drew upon and adapted selected content from an evidence‐based affirmative group‐based CBT intervention to reduce psychosocial distress and improve social support and coping among SGM youth in Canada, called AFFIRM [35, 38]. The research team combined the selected content (which focused on SGM identity‐affirming messages, and developing skills regarding CBT), and additional session content (which focused on addressing HIV‐related and intersectional stigma, violence, affirming human rights in the context of criminalization, building supportive networks and HIV services such as PrEP and treatment) from several other curricula [39, 40, 41]. Intersectional stigma was addressed by examining (and inviting contributions of) lived experiences in all their complexity, and structural factors that lead to these stigmas (e.g. discriminatory laws), with an emphasis on affirmative strategies to promote robust, shared resilience. The draft curriculum was pilot‐tested and refined in consultation with community members (peer educators/key opinion leaders [KOLs] with experience working with the SGM community around HIV and other topics).

### 2.2 Study design, sampling and recruitment

**Figure 1:**
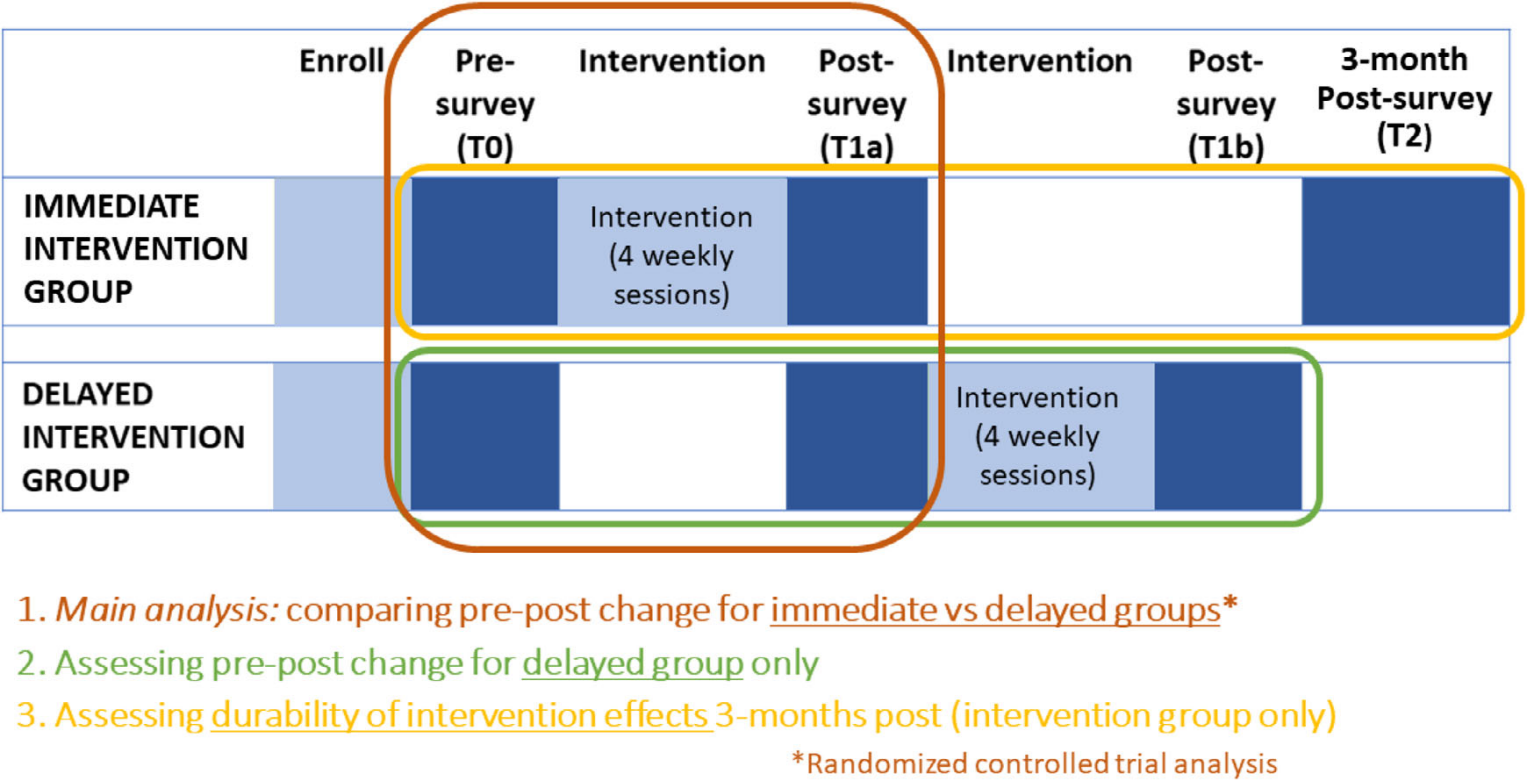
Study design.

We implemented a randomised controlled trial with immediate intervention and delayed intervention (comparison) groups (Figure 1), to ensure that all participants had the opportunity to access intervention activities. Eligible participants were at least 18 years old, willing to participate in all intervention sessions, and reported anticipating residing in Lagos for the next year, that they were assigned male at birth, and that they had sex with a biological man in the last year (for MSM participants) or self‐identified as a woman and had sex in the past year (for TGW participants). Participants were recruited through two strategies: (i) as existing clients at partner facilities; and (ii) by KOLs through their networks. To evaluate the acceptability and  preliminary efficacy of the intervention, we administered pre‐, post‐ and 3‐months post‐intervention surveys to participants. More details about power analysis and randomisation procedures are included in File S1. All three survey rounds were conducted by trained research assistants by telephone, and lasted approximately 20−25 minutes.

Our primary hypothesis (see Figure 1) was that respondents would report greater improvements in psychosocial outcomes (e.g. internalized stigma) in the immediate versus delayed group. We also hypothesized that the delayed group would show positive shifts in psychosocial outcomes between baseline and post‐intervention, and, that at 3‐months post‐intervention, the immediate group would report greater HIV service use (e.g. PrEP) than post‐delayed group, controlling for baseline levels, as well as sustained improvements in psychosocial outcomes (durability of intervention effects).

### 2.3 Outcomes and measures

Outcomes included internalized stigma related to SGM and HIV status (plus intersectional stigma), as well as depression, anxiety, coping and PrEP/HIV treatment use. For all continuous variables, the final possible score range was 0.0−3.0.

*Internalized stigma* was assessed by parallel sets of items related to MSM, TGW and HIV status, asked of each respective group of participants. Item examples include “I feel guilty that I have sex with men/am attracted to men,” “I deserve bad things happening to me because I have sex with men/am attracted to men” and “I try to hide the fact that I have sex with men/am attracted to men.” Response categories were strongly disagree/disagree/agree/strongly agree. These scales were adapted from several validated scales [42, 43, 44], particularly Kalichman et al.’s Internalized AIDS‐Related Stigma Scale [44], by two scale development experts [JP; AG] with deep experience related to stigma. Exploratory factor analysis using T0 (baseline) data identified two underlying factors (negative feelings, 6 items; disclosure, 3 items), and items within them that performed well across the three groups (MSM, TGW and HIV). Cronbach's alphas for each full 9‐item scale demonstrated good internal consistency reliability (IS‐MSM‐0.69; IS‐TGW‐0.75; IS‐HIV‐0.75). Final mean scores ranged from 0.0 to 3.0, with a higher score representing more stigma. We combined the MSM and TGW scales to measure stigma related to being a sexual and/or gender minority (SGM). Finally, we constructed an intersectional internalized stigma scale (i.e. SGM and HIV) by combining these two scale scores following a geometric approach (a^2^ +b^2^ = c^2^) [45].

*Depression* was assessed using the Patient Health Questionnaire‐8 with eight items assessing depression such as having “little interest or pleasure in doing things,” and feeling “down, depressed or hopeless” [46]. *Anxiety* was assessed using the General Anxiety Disorder‐7, such as “trouble relaxing” and “not being able to control or stop worrying” [47]. Both scales are asked with a reference period of the last 2 weeks. *Coping* was measured using the Brief Coping Scale, which includes four items [48].

HIV testing in the last 6 months and current PrEP use were self‐reported binary measures, assessed among participants not reporting being a person with HIV at any round. Among those with HIV, adherence was defined as reporting “No days” to the question “During the past 3 days, on how many days have you missed all your pills?” [49, 50]. Antiretroviral *treatment self‐efficacy* was assessed via the mean score (1−5) on seven items from a validated scale [51] with last month as the reference period.

### 2.4 Qualitative research

We conducted qualitative research with a subset of participants 1 month after the final survey, to further understand experiences with the intervention as well as help elucidate possible reasons for selected quantitative findings emerging from the initial survey analysis. To capture potentially heterogeneous intervention participant perspectives and generate an information‐rich, sufficiently saturated sample [52], 24 IDIs were conducted (six MSM and six TGW from each of the intervention and delayed groups). We also conducted IDIs with the two programme managers who oversaw intervention implementation, and the counsellor/psychologist who provided individual counselling to participants when requested. In addition, we held an FGD with all CHEWs who facilitated the group sessions (*n* = 5). More details about qualitative research methods are included in File S1.

### 2.5 Data analysis

#### 2.5.1 Quantitative analysis

All analyses were conducted in Stata v16 (StataCorp LLC, College Station, TX). Statistical comparisons of baseline characteristics by study arm were assessed via bivariate logistic/linear regression. Assessments of intervention effects on psychosocial outcomes (all continuous measures) were conducted using mixed model repeated measures, a common analytic approach for randomised controlled trial (RCTs) with more than two time points (which can also accommodate analyses at only two time points). For intent‐to‐treat (ITT) analyses to test intervention effects (T0 vs. T1a), estimated coefficients included the effect of time, and the effect of time × trial arm (ITT effect), controlling for baseline age, education, how recruited, interviewer, MSM/TGW status, relationship status, ability to meet basic needs, employment status and HIV status. We did not conduct subgroup nor effect modification by MSM versus TG subgroups, nor by intervention group‐session membership, since our study was not powered to do so.

To assess change over time in psychosocial outcomes, we estimated coefficients comparing outcomes between relevant time points, controlling for the characteristics noted above. For analyses of differences in HIV service use at T2 in the immediate group versus T1b in the delayed group, we conducted logistic or linear regression estimating the effect of trial arm, controlling for baseline service use levels and the characteristics noted above. Current PrEP use was not captured at baseline, thus for related analyses, we controlled for reported baseline ever‐use of PrEP instead.

#### 2.5.2 Qualitative data analysis

Interviews were transcribed/translated, checked for accuracy and entered into NVivo 12 for coding. Thematic analysis was used to identify salient themes and patterns. More details about qualitative analysis methods are included in File S1.

## 3 RESULTS

Out of 306 people assessed for eligibility, 24 refused to participate and 44 did not meet inclusion criteria, resulting in 204 individuals being randomised to either the immediate (*n* = 117) or delayed arm (*n* = 123). Two people were lost to follow‐up in the immediate arm, and one person in the delayed arm. Of the 240 participants at baseline (T0), there were 185 MSM and 55 TGW.

Table 1 shows sample characteristics at baseline. There were no differences by study arm. Overall, respondents were 26 years old on average (range 18−42 years). About three‐quarters (77%) self‐identified as MSM, and one‐quarter as TGW (asked for purposes of participating in MSM or TGW‐specific sessions). Over one‐third (37%) reported being diagnosed with HIV at all study rounds. Among respondents with HIV, about 10% had known their HIV status for 1 year or less, 40% for 1−2 years (inclusive), 30% 3−5 years (inclusive) and the remaining 20% for 6−13 years (mean years since diagnosis is included in Table 1). About half (54%) had completed at least university/trade school (vs. high school or less). Over half (about 61%) reported being employed, most with informal/casual salary earnings. Only 12% reported being able to meet their basic needs “most of the time,” with 80% saying “some of the time” and 8% “never.”

**Table 1:** Sample characteristics at baseline (overall and by study arm) and intervention participation

	Overall (*n* = 240)	Immediate group (*n* = 117)	Delayed group (*n* = 123)	Beta/Odds ratio for difference (immediate vs. delayed)
**Age in years** (SD, range)	26.0 (4.5, 18−42)	25.9 (4.7, 18−38)	26.1 (4.4, 18−42)	−0.23 (−1.38, 0.93)
**Self‐identification for intervention purposes**				
MSM	185 (77.1%)	95 (81.2%)	90 (73.2%)	1.58 (0.86, 2.92)
Transgender woman	55 (22.9%)	22(81.2%)	33 (26.8%)	
**Diagnosed with HIV** (vs. HIV negative/status unknown; at all study rounds)	89 (37.1%)	46 (39.3%)	43 (35.0%)	1.21 (0.71, 2.04)
**Years since HIV diagnosis—**mean (SD, range)	3.71 (3.00, 0−15)	3.81 (3.32, 0−15)	3.63 (2.69, 0−11)	−0.18 (−1.45, 1.09)
**Relationship status**				
Not in a relationship	173 (72.1%)	84 (71.8%)	89 (72.4%)	1.03 (0.58, 1.81)
In a relationship but not living together	59 (24.6%)	28 (23.9%)	31 (25.2%)	
In a relationship and living together	8 (3.3%)	5 (4.3%)	3 (2.4%)	
**Education** (Highest achieved—University/trade school vs. high school or less)	129 (53.8%)	56 (47.9%)	73 (59.4%)	0.63 (0.38, 1.05)
**Employment status**				
No, and not looking for work	47 (19.6%)	21 (18.0%)	26 (21.1%)	1.23 (0.65, 2.32)
No, but looking for work	46 (19.2%)	19 (16.2%)	27 (22.0%)	
Yes, informal/casual salary earnings	133 (55.4%)	69 (59.0%)	64 (52.0%)	
Yes, formal salary/wages	14 (5.8%)	8 (6.8%)	6 (4.9%)	
**Inability to meet basic needs** ^a^				
Never	18 (7.5%)	11 (9.4%)	7 (5.7%)	0.58 (0.22, 1.55)
Some of the time	193 (80.4%)	92 (78.6%)	101 (82.11%)	
Most of the time	29 (12.1%)	14 (12.0%)	15 (12.2%)	
**Alcohol abuse/hazardous drinking**				
Mean score^b^; possible range 0−12 (SD, range)	1.15 (1.41, 0−7)	1.27 (1.41, 0−6)	1.06 (1.40, 0−7)	−0.21 (−0.15, 0.57)
**Mode of recruitment**				
Key opinion leader or Community‐based organization staff	150 (62.5%)	77 (65.8%)	73 (59.4%)	1.32 (0.78, 2.23)
A friend	90 (37.5%)	40 (34.2%)	50 (40.7%)	
**Number of sessions attended** (out of 4)	(*n* = 208^c^)	(*n* = 87)	(*n* = 121)	
4	183 (88.0%)	76 (87.4%)	107 (88.4%)	−0.15 (−0.99, 0.69)
3	19 (9.1%)	6 (6.9%)	13 (10.7%)	
2	4 (1.9%)	4 (4.6%)	0 (0%)	
1	2 (1.0%)	1 (1.2%)	1 (0.8%)	

*Note*: All comparisons between trial arms were not statistically significant, at *p*<0.05.

Abbreviations: MSM, men who have sex with men; SD, standard deviation; TGW, transgender women.

^a^ Question wording was “In the last 12 months, how often have you been unable to meet basic needs (e.g., food, shelter, clothing)?”

^b^ Mean score on the Alcohol Use Disorders Identification Test (AUDIT‐C), a validated 3‐item screening tool.

^c^ Thirty‐two respondents were missing data due to a survey coding error.

Nearly two‐thirds of participants were recruited by KOLs/CBO staff and one‐third by friends. Most (88%) of participants attended all four workshop sessions.

Table 2 shows scores on each of the psychosocial outcome variables, by trial arm at each time point. There were no differences between the immediate and delayed arms at baseline (T0), at *p*<0.05. As can be seen, both the immediate and delayed intervention arms followed a similar pattern/trajectory of improvements over time in most cases.

**Table 2:** Psychosocial outcomes at each study time point, in immediate and delayed arms

	Group	*N*	T0	T1a	T1b	T2
**IS‐SGM**	Immediate	117	1.23	0.99	−	1.16
	Delayed	123	1.19	0.94	1.12	−
**IS‐HIV**	Immediate	46	1.48	1.28	−	1.37
	Delayed	43	1.48	1.16	1.32	−
**Intersectional IS**	Immediate	46	1.41	1.17	−	1.27
	Delayed	43	1.34	1.09	1.24	−
**Depression**	Immediate	117	0.76	0.37	−	0.15
	Delayed	123	0.75	0.35	0.25	−
**Anxiety**	Immediate	117	0.81	0.41	−	0.13
	Delayed	123	0.77	0.39	0.25	−
**Coping**	Immediate	117	2.20	2.25	−	2.35
	Delayed	123	2.15	2.36	2.28	−

*Note*: The possible range of all variables is 0.0−3.0.

Abbreviations: IS, internalized stigma; SGM, sexual and/or gender minority.

Table 3 includes the adjusted ITT effects (difference‐in‐difference analysis), comparing change in psychosocial variables in the immediate intervention and delayed intervention (i.e. control) arms, limited to T0 and T1a time points. There were significant improvements in each psychosocial outcome between the two time points. However, these positive shifts were found in both the immediate and delayed arms, and thus we did not detect an intervention effect.

**Table 3:** Intent‐to‐treat effects

	*N*	Effect of round (T0−T1a) (adj. Beta)	*p*‐value	Intervention effect (round*trial arm) (adj. Beta)	*p*‐value
**IS‐SGM**	240	−0.245	0.000	0.007	0.914
**IS‐HIV**	129	−0.348	0.000	0.160	0.088
**Intersectional IS**	129	−0.284	0.000	0.052	0.513
**Depression**	240	−0.403	0.000	0.012	0.852
**Anxiety**	240	−0.380	0.000	−0.030	0.675
**Coping**	240	0.211	0.000	−0.160	0.054

*Note*: *Controls (all measured at baseline)*: age, education, how recruited, interviewer, MSM/TGW per intervention participation, relationship status, ability to meet basic needs and employment status (as well as HIV status, for models not limited to people with HIV).

Abbreviations: IS, internalized stigma; SGM, sexual and/or gender minority.

These positive shifts were further improved for some variables (e.g. depression) at 3 months post‐intervention for the immediate group (T2). (Note that there was no 3‐month follow‐up for the delayed group.) Table 4 shows the immediate group changes over time. Specifically, comparing between T0 and T2 (baseline and 3‐months post‐intervention), there were improvements in intersectional stigma, depression, anxiety and coping. Comparing between T1a and T2 (immediately after intervention and 3‐months post‐intervention), there were further improvements in depression and anxiety, but a drop‐off effect for IS‐SGM and intersectional IS (where levels rebounded, although not to baseline levels, after initially improving between baseline and immediately after the intervention).

**Table 4:** Immediate group change over time (durability of effect)

	*N*	Effect of T2 (comparing to T0)	*p*‐value	Effect of T2 (comparing to T1a) *Durability*	*p*‐value
IS‐SGM	117	−0.071	0.058	0.166 (unanticipated direction)	<0.001
IS‐HIV	66	−0.080	0.175	0.116	0.053
Intersectional IS	66	−0.104	0.019	0.140 (unanticipated direction)	0.009
Depression	117	−0.615	<0.001	−0.225	<0.001
Anxiety	117	−0.686	<0.001	−0.278	<0.001
Coping	117	0.141	0.004	0.090	0.128

*Note*: *Controls (all measured at baseline)*: age, education, how recruited, interviewer, MSM/TGW per intervention participation, relationship status, ability to meet basic needs and employment status (as well as HIV status, for models not limited to people with HIV).

Abbreviations: IS, internalized stigma; SGM, sexual and/or gender minority.

Results related to change over time for the delayed group were mixed (Table S1). Specifically, comparing between T0 and T1b (baseline and post‐intervention), there were improvements in IS‐HIV, depression, anxiety and coping. Comparing between T1a and T1b (pre‐post intervention), there were improvements in depression and anxiety, but a rebound of each type of internalized stigma (although no higher than at baseline).

Table 5 shows HIV testing and current PrEP use among respondents not reporting being diagnosed with HIV, and ART adherence and self‐efficacy among those with HIV. We report baseline levels and those at the final time point for each group. We compare between T2 at 3‐months post‐intervention for the immediate group, to T1b right after the intervention for the delayed group, effectively treating the delayed group as a control since realistically it would take time for participants to take up HIV services after completing the intervention. Nearly three‐quarters (73%) of immediate group respondents reported current PrEP use at 3 months after completing the intervention, versus half (52%) of delayed group participants right after they completed the intervention. This represents 4.4 times the odds of current PrEP use (controlling for baseline levels of ever PrEP use which did not differ between trial arms, as well as other socio‐demographic characteristics; *p*<0.01). HIV testing was also higher in the immediate group, but this did not reach statistical significance (*p* = 0.1). Among respondents with HIV, we note that all respondents reported current ART use and thus there was no potential for improvement (NS). There was no effect on adherence or treatment self‐efficacy.

**Table 5:** HIV service use at 3‐month post in immediate group versus direct‐post in delayed group, controlling for baseline levels

	Trial arm	% at T0	% at T2 3 months post‐intervention (for immediate group) or T1b immediately after intervention (for delayed group)	aOR/adj Beta for immediate versus delayed group^a^
**HIV testing in last 6 months** ^b^	Immediate group (*n* = 51)	84.3%	96.0%	3.42 (0.78, 15.02)
	Delayed group (*n* = 48)	83.3%	91.7%	
**Current PrEP use** ^b^	Immediate group (*n* = 51)	62.8% ever used PrEP	72.6%	4.36 (1.57, 12.15)^**^
	Delayed group (*n* = 48)	70.8% ever used PrEP	52.1%	
**Adherence to antiretroviral therapy** ^c^	Immediate group (*n* = 46)	73.91%	89.13%	1.35 (0.32, 5.72)
	Delayed group (*n* = 43)	83.7%	88.37%	
**Treatment self‐efficacy** ^c^ (possible range: 1.0−5.0)	Immediate group (*n* = 46)	4.121	4.016	−0.12 (−0.41, 0.17)
	Delayed group (*n* = 43)	4.063	4.040	

Abbreviation: PrEP, pre‐exposure prophylaxis.

^a^ Adjusted odds of the outcome in Immediate group at 3‐month post compared with delayed group at immediate post, controlling for T0 (for current PrEP use outcome, controlled for ever‐PrEP use at T0), as well as *Controls (all measured at baseline)*: age, education, how recruited, interviewer, MSM/TGW per intervention participation, relationship status, ability to meet basic needs and employment status. Adherence and treatment literacy models also controlled for length of time knowing HIV status.

^b^ Among those never reporting being diagnosed with HIV at any round.

^c^ Among those reporting being diagnosed with HIV at all rounds, for comparability. Note all respondents reported currently taking ART at each time point.

^**^ *p*<0.01.

**Figure 2:**
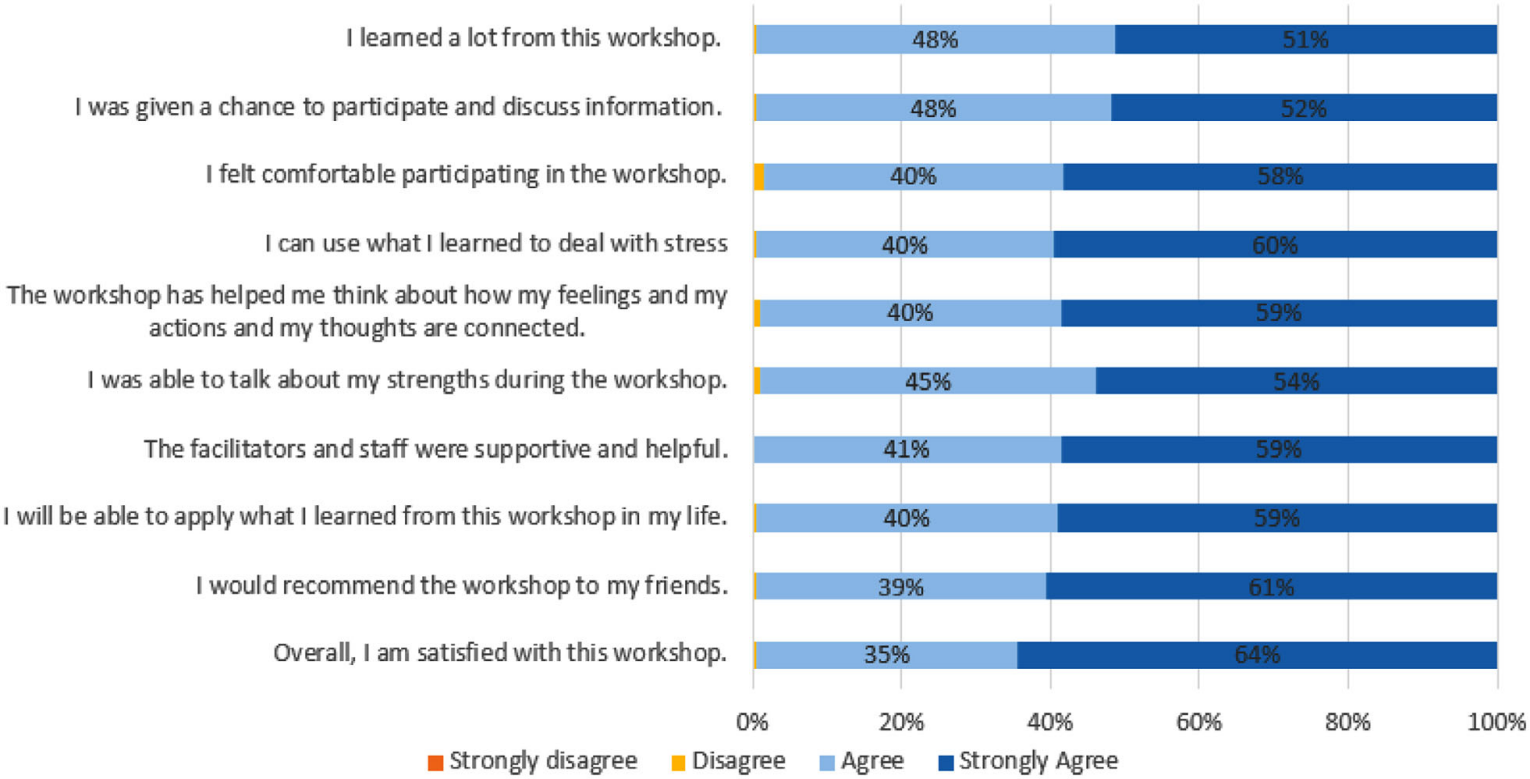
Reported satisfaction with the intervention. Among both immediate and delayed groups combined; reported satisfaction did not significantly differ between arms. Satisfaction questions were asked at the survey immediately following intervention completion (T1a for immediate group and T1b for delayed group). *n* = 208; 32 respondents were missing data due to a survey coding error.

Figure 2 shows reported satisfaction with the intervention, pooled across immediate and delayed group participants (noting responses were similar in each). Over 98% of participants agreed or strongly agreed with each statement representing satisfaction with different aspects of the workshop, with most strongly agreeing with each statement, especially “I would recommend the workshop to my friends” (61% strongly agreed) and “Overall, I am satisfied with this workshop” (64% strongly agreed).

### 3.1 Qualitative findings

The median age of participants from the 24 IDIs was 25.5 years for TGW participants and 23.0 years for MSM participants. Half were diagnosed with HIV. There were several themes that emerged from the data related to positive intervention influence on psychosocial wellbeing and HIV service uptake, in addition to possible reasons for improvements reported in psychosocial outcomes in the delayed group despite not yet having participated in the workshops.

### 3.2 Intervention influence on psychosocial wellbeing and HIV service uptake

In the qualitative interviews, participants consistently expressed that the intervention led to their feeling more self‐confident and less self‐blame about their sexual and gender identities, and that the sessions produced a safe space where they could feel supported and could support one another. For example, facilitators of the programme noted that each small group had formed WhatsApp groups via which they continued to connect with each other since the intervention.

Many participants also reported that the programme enabled them to take up and/or adhere to HIV services, such as HIV testing, PrEP and viral load tracking. For example, several participants described initiating (or re‐initiating) PrEP because of the programme. In general, participants described two main reasons why the intervention supported HIV service uptake. First, because the intervention led to less internalized stigma and more resiliency and social support, which in turn enabled service use. Second, many participants reported that the workshops provided the time and welcoming space needed to learn about the available prevention and treatment/care options and services and how they support wellbeing. Examples of these themes are shared in Table 6.

**Table 6:** Quotes reflecting key themes about effects of intervention

Theme	Quotes
*Reduced internalized stigma*	*Before your workshop, ah, I used to think so bad about myself…I just hated myself but after the workshop and everything, I was like yes, I'm still a somebody…we will be fine and we will cope, so life continue*. ***(TGW, Immediate intervention group)***
	*You know, being a queer guy is a very heavy burden on its own. Then getting to find out that you are HIV positive, and contacting it through like queer sex, it really had a very, it had a hard toll on me…During the workshop session, I got to find out that I can just still be anything I want to be and still feel the same way normal people that are not positive still feel. (* ***MSM, Immediate intervention group)***
*Resiliency and coping*	*The workshop opened my eyes to so many things sha, that if I don't take care of myself, no one else will, and if it has happened, it has happened…I just need to know how to deal with it and necessary steps to take and how to be a human being….I had to just rush down to collect the ARV because I was like okay, there is more to life than, so, to like bothering myself that I am positive or let it get to me…So, the workshop helped me*. ***(MSM, Immediate intervention group)***
*Welcoming space needed for learning about PrEP/HIV prevention*	*I've heard of PrEP, but most of the things I was reading online didn't allow me to okay, fully be consistent with it but when I came to the workshop…I think that's made me adjust…we're able to ask questions, we were enlightened more about the situation and that really helped. (* ***MSM, Immediate intervention group)***
	*Respondent: I must confess, uh, before now I don't take PrEP, I see like punishment. But after this study, now that you told me more about PrEP, I usually take it normally the way I'm supposed to*….*Yes, like two of my friends feel that they don't use PrEP before [but] they started taking it through because of the workshop*. ***(TGW, Immediate intervention group)***
	*they spoke about adherence as an effective coping mechanism…He also gave them an insight on what PrEP really is and how to use this effectively. And then we noticed an increase in PrEP uptake by most of the negative participants, a lot of them went for it*. ***(Community Mobilizer)***
	*The thing is that when, when I started this survey, I don't know anything about viral suppression, so it was, it was when she explained more better about it, so I get to know that this is what I'm meant to do*. ***(TGW, Delayed intervention group)***

Abbreviations: ARV, antiretroviral medications; MSM, man who has sex with men; PrEP, pre‐exposure prophylaxis; TGW, transgender woman.

### 3.3 Possible reasons for shifts in both the immediate and delayed intervention groups

We also explored possible reasons for improvements reported in psychosocial outcomes in the delayed group despite not yet having participated in the workshop. The responses of participants and programme staff suggested there was a mix of reasons. Most commonly, these included a positive reaction to the study team during enrolment and the surveys, and to having the opportunity to talk about sensitive issues for the first time, as well as anticipation of soon participating in workshops on these important topics (again often for the first time). Respondents also described hearing positive feedback about the workshops and some limited content from participants in the immediate intervention group (which could have led to some “contamination” between the two groups). Others referred to participating in Pride month activities (mainly online awareness raising/advocacy events, with some covert in‐person parties) that were ongoing during the study period. Examples of these different reasons are included in Table 7.

**Table 7:** Quotes around possible explanations for quantitative evaluation results

Theme	Quotes
*Positive interactions with the study team*	Interviewer: *So, do you think it's the participation in the first [survey] that made people to feel better before the second survey or even before the workshop?* Respondent: *Personally, it helped me in a way because there's something that happens when you get to talk about your challenges or your struggles even without solving them, talking about it alone has a way of relieving you. So, I think it helped*. ***(MSM, Delayed intervention group)***
	*[The research assistant] was asking… the personal questions about yourself…I just gave him like a hundred percent um, confidential information about myself, then I felt he's a person I can relate with. Even after the phone conversation we still talk on WhatsApp and other platforms to talk about more about yourself, “this is what I'm facing Mr. ___.”* ***(TGW, Delayed intervention group)***
	*…knowing that there's a study that is you know that is going on, you know, teach them how to cope life even more, so them knowing that they will attend and know, it will make them feel better…* ***(TGW, Delayed intervention group)***
*Learning about workshops from previous participants*	*He just told me about the teachings that, um, PC organized the session and has changed his life. And I was like, wow, how, what's it, what, what is it all about?* ***(MSM, Delayed intervention group)***
	*He told me about the study he told me that they taught them about stigma how to cope with life…so it's one of things that motivated* ***(TGW, Delayed intervention group)***
*Influence of Pride celebrations*	*Once it's month of June…even in Nigeria, in as much as that gay is not legalized in Nigeria, they always feel kind of free…they are always happy during that month because believe that this is their month*. ***(TGW, Delayed intervention group)***
	*There was nothing like Pride Month, but now there is, and it's just us telling the world, “We are here”…because people still don't wrap their head around the fact that we actually have like gay men or trans people living in Nigeria* ***(MSM, Delayed intervention group)***

Abbreviations: MSM, man who has sex with men; PC, Population Council; TGW, transgender woman.

## 4 DISCUSSION

Results from this rigorous evaluation of a novel group CBT‐based intervention to reduce internalized stigma and support mental health and HIV wellness for MSM and TGW at risk for/diagnosed with HIV in Lagos, Nigeria demonstrated near‐universal programme participation and very high participant satisfaction. We also found marked improvements in psychosocial outcomes—including internalized stigma and depression—among both respondents who had received, and not yet received, the intervention, which indicates that we cannot conclude that the improvements were due to the intervention per the RCT design. Yet, when triangulating with other study data points, there are some positive initial results. At 3‐month post participation for the immediate intervention group, there were further improvements (e.g. in depression and coping). PrEP comparisons at the endline suggested an intervention effect on uptake. And findings from IDIs with participants (and programme staff) suggested positive effects of the intervention such as skills and support networks developed that could be applied to reduce internalized stigma and promote resilience, and provided insights as to why there were positive changes in the delayed group as well.

Putting this study in context within the existing literature, internalized stigma has been identified as strongly and consistently associated with poor health outcomes [53]. Advocates have highlighted the urgent need for more evidence around HIV programming that can address internalized stigma, including by integrating more intersectional conceptions of stigma [16]. For such efforts, the literature suggests that partnering with communities/CBOs (community‐based organization) that maintain trusted spaces, using identity‐affirming practices, and applying an approach that addresses HIV self‐care as part of mental and physical “wellness” more broadly—as we did in the current study—are important ways to incorporate an intersectional lens into programming [54]. While there are limited publications available related to programming for SGMs that address stigma in the African setting, a recent systematic review of 37 health interventions for SGMs (focused on the United States) concluded that an intersectional framework was needed but usually absent in such interventions, and no studies included measures for intersectional stigma [55]. To contribute to measurement in this area, for the current study, we adapted existing stigma measures to capture internalized stigma with an intersectional lens—resulting in well‐performing scales with good internal consistency reliability.

Our study applied CBT‐based strategies to address internalized stigma and mental health among the SGM community in Nigeria. The literature on CBT in Africa is limited but growing. For example, a pilot study using CBT was successfully conducted in South Africa to address depression and ART adherence among people with HIV [56], suggesting that issues like HIV stigma could be appropriate for such an intervention. A 2022 book describing the global use of CBT, including in Africa, highlighted that replication and implementation studies were still needed to strengthen the evidence base [57].

Regarding the positive psychosocial shifts seen between baseline and start of the delayed intervention, respondents described benefits simply from participating in the two surveys (each with the same interviewer with whom they had developed a rapport) and from being invited for the first time to participate in a stigma and mental health programme. Implications include that one‐on‐one interventions that are not necessarily conducted by mental health professionals yet are less intensive than the four‐session programme could also be beneficial for some outcomes. Future research could test the pros and cons of these options.

Limitations of the study include that there was no opportunity to add a 3‐month post‐test to the delayed intervention, so that this could be compared with the 3 months post‐test after the immediate intervention. It was also difficult to take into account the effects of external activities such as those taking place during Pride month, although likely they would have equally affected both groups. It was also not possible to clearly ascertain whether the reported uptake in HIV services, in particular PrEP, was due to reductions in internalized stigma.

Other prevalence studies with MSM and TGW in Nigeria suggest that the population included in the current study is relatively comparable to the larger SGM community, and thus findings could be applied to the larger community [23, 58]. However, further research would still be needed to confirm the effects of this intervention. For example, a follow‐up study with more people without HIV to confirm PrEP uptake, and/or with larger sub‐samples of persons with HIV to permit sufficient power for sub‐analyses, would be two useful directions. Implementation science outcomes (e.g. fidelity, cost) could also be utilized in future studies to help evaluate effectiveness.

Some recommendations can already be derived from the programme development and study implementation experience. The group format and use of community‐based facilitators permit reaching a larger audience than one‐on‐one counselling, which is a strength in settings with limited access to mental health providers; however, given the programme time commitment involved, participants should be offered options for the timing of the sessions (as was done for this study). Regarding measurement, with shifting recommended terminology over time [59] for terms such as “MSM” and “HIV‐positive,” it will be important to revisit survey/scale items when applying them in the future to ensure that they are not further stigmatizing. Content‐wise, such an intervention openly addressing issues of stigma and mental health was sorely needed in this context, as shared by participants. There are numerous locations across the globe where the LGBTQ+ community is criminalized and/or stigmatized, suggesting the potential widespread application of this type of intervention.

## 5 CONCLUSIONS

This study demonstrated the feasibility and acceptability of a group‐based CBT model to reduce stigma and support mental health among MSM and TGW in Nigeria. Findings derived from this mixed methods study provide some indications of positive shifts related to mental health, stigma, and PrEP motivation and uptake (despite the challenges with the RCT design). Given the critical need for HIV wellness and mental health promotion programmes for SGMs and other marginalized populations in Nigeria—and elsewhere—these initial results point to a promising option.

## COMPETING INTERESTS

The authors do not have any competing/conflicts of interest.

## AUTHORS’ CONTRIBUTIONS

JP, WT, AG and AA designed the study. AG led and JP, WT, AFE and PLO supported intervention/curriculum development. AFE, PLO, ES and MA supported study implementation such as via intervention facilitation or the recruitment of participants. AG and WT led the analyses, with JP and AA contributing to the analysis plan and interpretation. JP led, and AG, WT and AA contributed to the writing of the manuscript. All authors reviewed and approved the final manuscript.

## FUNDING

Funding for this project was made available by the Elton John AIDS Foundation.

## Supporting information

Supplemental File 1. Additional details regarding study methods.

Appendix Table S1. Delayed group changes over time.

## Data Availability

The de‐identified dataset and surveys can be requested through the Population Council Dataverse (https://dataverse.harvard.edu/dataverse/popcouncil).

## References

[1] Adebajo SB , Eluwa GI , Allman D , Myers T , Ahonsi BA . Prevalence of internalized homophobia and HIV associated risks among men who have sex with men in Nigeria. Afr J Reprod Health. 2012;16(4):21–28. 23444540

[2] Geibel S , Gottert A , Friedland BA , Jeremiah K , McClair TL , Mallouris C , et al. Internalized stigma among people living with HIV: assessing the Internalized AIDS‐Related Stigma Scale in four countries. LWW; 2020. 10.1097/QAD.0000000000002649 32881792

[3] Kane JC , Elafros MA , Murray SM , Mitchell EM , Augustinavicius JL , Causevic S , et al. A scoping review of health‐related stigma outcomes for high‐burden diseases in low‐ and middle‐income countries. BMC Med. 2019;17(1):1–40. 30764819 10.1186/s12916-019-1250-8 PMC6376728

[4] King R , Nanteza J , Sebyala Z , Bbaale J , Sande E , Poteat T , et al. HIV and transgender women in Kampala, Uganda–Double Jeopardy. Cult Health Sex. 2019;21(6):727–740. 30328785 10.1080/13691058.2018.1506155

[5] Mbeda C , Ogendo A , Lando R , Schnabel D , Gust DA , Guo X , et al. Healthcare‐related stigma among men who have sex with men and transgender women in sub‐Saharan Africa participating in HIV Prevention Trials Network (HPTN) 075 study. AIDS Care. 2020;32(8):1052–1060. 32500722 10.1080/09540121.2020.1776824 PMC7368806

[6] Oginni OA , Mapayi BM , Afolabi OT , Obiajunwa C , Oloniniyi IO . Internalized homophobia, coping, and quality of life among Nigerian gay and bisexual men. J Homosex. 2020;67(10):1447–1470. 30977714 10.1080/00918369.2019.1600899

[7] Poteat T , Ackerman B , Diouf D , Ceesay N , Mothopeng T , Odette K‐Z , et al. HIV prevalence and behavioral and psychosocial factors among transgender women and cisgender men who have sex with men in 8 African countries: a cross‐sectional analysis. PLoS Med. 2017;14(11):e1002422. 29112689 10.1371/journal.pmed.1002422 PMC5675306

[8] Tun W , Pulerwitz J , Shoyemi E , Fernandez A , Adeniran A , Ejiogu F , et al. A qualitative study of how stigma influences HIV services for transgender men and women in Nigeria. J Int AIDS Soc. 2022;25:e25933. 35818868 10.1002/jia2.25933 PMC9274359

[9] Sievwright KM , Stangl AL , Nyblade L , Lippman SA , Logie CH , de Sousa Mascena Veras MA , et al. An expanded definition of intersectional stigma for public health research and praxis. American Public Health Association; 2022. 10.2105/AJPH.2022.306718 PMC9241457 35763723

[10] Lyons CE , Olawore O , Turpin G , Coly K , Ketende S , Liestman B , et al. Intersectional stigmas and HIV‐related outcomes among a cohort of key populations enrolled in stigma mitigation interventions in Senegal. AIDS. 2020;34(Suppl 1):S63. 32881795 10.1097/QAD.0000000000002641 PMC7880051

[11] Thompson EC , Muhammad JN , Adimora AA , Chandran A , Cohen MH , Crockett KB , et al. Internalized HIV‐related stigma and neurocognitive functioning among women living with HIV. AIDS Patient Care STDs. 2022;36(9):336–342. 36099481 10.1089/apc.2022.0041 PMC9810353

[12] Tsai AC , Burns BF . Syndemics of psychosocial problems and HIV risk: a systematic review of empirical tests of the disease interaction concept. Soc Sci Med. 2015;139:26–35. 26150065 10.1016/j.socscimed.2015.06.024 PMC4519429

[13] Viswasam N , Schwartz S , Baral S . Characterizing the role of intersecting stigmas and sustained inequities in driving HIV syndemics across low‐ and middle‐income settings. Curr Opin HIV AIDS. 2020;15(4):243. 32487815 10.1097/COH.0000000000000630 PMC7875118

[14] Dale SK , Ayala G , Logie CH , Bowleg L . Addressing HIV‐related intersectional stigma and discrimination to improve public health outcomes: an AJPH supplement. American Public Health Association; 2022. 10.2105/AJPH.2022.306738 PMC9241474 35763724

[15] Livingston JD , Boyd JE . Correlates and consequences of internalized stigma for people living with mental illness: a systematic review and meta‐analysis. Soc Sci Med. 2010;71(12):2150–2161. 21051128 10.1016/j.socscimed.2010.09.030

[16] Pantelic M , Sprague L , Stangl AL . It's not “all in your head”: critical knowledge gaps on internalized HIV stigma and a call for integrating social and structural conceptualizations. BMC Infect Dis. 2019;19(1):1–8. 30832613 10.1186/s12879-019-3704-1 PMC6399894

[17] Crowell TA , Lawlor J , Lombardi K , Nowak RG , Hardick J , Odeyemi S , et al. Anorectal and urogenital *Mycoplasma genitalium* in Nigerian men who have sex with men and transgender women: prevalence, incidence, and association with HIV. Sex Transm Dis. 2020;47(3):202. 31880740 10.1097/OLQ.0000000000001105 PMC7309576

[18] Jones J , Sanchez TH , Dominguez K , Bekker LG , Phaswana‐Mafuya N , Baral SD , et al. Sexually transmitted infection screening, prevalence and incidence among South African men and transgender women who have sex with men enrolled in a combination HIV prevention cohort study: the Sibanye Methods for Prevention Packages Programme (MP3) project. J Int AIDS Soc. 2020;23:e25594. 33000886 10.1002/jia2.25594 PMC7527766

[19] Kimani M , van der Elst EM , Chiro O , Oduor C , Wahome E , Kazungu W , et al. Pr EP interest and HIV‐1 incidence among MSM and transgender women in coastal Kenya. J Int AIDS Soc. 2019;22(6):e25323. 31194291 10.1002/jia2.25323 PMC6563853

[20] Sandfort TG , Mbilizi Y , Sanders EJ , Guo X , Cummings V , Hamilton EL , et al. HIV incidence in a multinational cohort of men and transgender women who have sex with men in sub‐Saharan Africa: findings from HPTN 075. PLoS One. 2021;16(2):e0247195. 33630925 10.1371/journal.pone.0247195 PMC7906338

[21] Leddy AM , Weiss E , Yam E , Pulerwitz J . Gender‐based violence and engagement in biomedical HIV prevention, care and treatment: a scoping review. BMC Public Health. 2019;19:1–14. 31286914 10.1186/s12889-019-7192-4 PMC6615289

[22] Peitzmeier SM , Malik M , Kattari SK , Marrow E , Stephenson R , Agénor M , et al. Intimate partner violence in transgender populations: systematic review and meta‐analysis of prevalence and correlates. Am J Public Health. 2020;110(9):e1–e14. 10.2105/AJPH.2020.305774 PMC7427218 32673114

[23] Federal Ministry of Health of Nigeria . Integrated Biological and Behavioural Surveillance Survey (IBBSS). 2020.

[24] Scheim AI , Santos GM , Arreola S , Makofane K , Do TD , Hebert P , et al. Inequities in access to HIV prevention services for transgender men: results of a global survey of men who have sex with men. J Int AIDS Soc. 2016;19:20779. 27431466 10.7448/IAS.19.3.20779 PMC4949311

[25] Daniel C , Ekanem E , Njab J , Oridota E , Robert A . Retention in care among HIV infected men who have sex with men attending a Community Health Centre, Yaba Lagos, Nigeria. J AIDS Clin Res. 2018;9(770):2.

[26] Schwartz SR , Nowak RG , Orazulike I , Keshinro B , Ake J , Kennedy S , et al. The immediate effect of the Same‐Sex Marriage Prohibition Act on stigma, discrimination, and engagement on HIV prevention and treatment services in men who have sex with men in Nigeria: analysis of prospective data from the TRUST cohort. Lancet HIV. 2015;2(7):e299–e306. 26125047 10.1016/S2352-3018(15)00078-8 PMC4481876

[27] Same Sex Marriage (Prohibition) Act, 2013.

[28] Pulerwitz J , Michaelis A , Weiss E , Brown L , Mahendra V . Reducing HIV‐related stigma: lessons learned from Horizons research and programs. Public Health Rep. 2010;125(2):272–281. 20297756 10.1177/003335491012500218 PMC2821857

[29] Duby Z , Fong‐Jaen F , Nkosi B , Brown B , Scheibe A . ‘We must treat them like all the other people’: evaluating the integrated key populations sensitivity training programme for healthcare workers in South Africa. South Afr J HIV Med. 2019;20(1):1–7. 10.4102/sajhivmed.v20i1.909 PMC6556945 31205777

[30] Logie CH , Dias LV , Jenkinson J , Newman PA , MacKenzie RK , Mothopeng T , et al. Exploring the potential of participatory theatre to reduce stigma and promote health equity for lesbian, gay, bisexual, and transgender (LGBT) people in Swaziland and Lesotho. Health Educ Behav. 2019;46(1):146–156. 29589481 10.1177/1090198118760682 PMC7025806

[31] Poteat T , Park C , Solares D , Williams JK , Wolf RC , Metheny N , et al. Changing hearts and minds: results from a multi‐country gender and sexual diversity training. PLoS One. 2017;12(9):e0184484. 28926568 10.1371/journal.pone.0184484 PMC5604941

[32] Butler AC , Chapman JE , Forman EM , Beck AT . The empirical status of cognitive‐behavioral therapy: a review of meta‐analyses. Clin Psychol Rev. 2006;26(1):17–31. 16199119 10.1016/j.cpr.2005.07.003

[33] Dobson KS , McEpplan AM , Dobson D . Empirical validation and the cognitive‐behavioral therapies. Guilford Press; 2019.

[34] Murray LK , Dorsey S , Haroz E , Lee C , Alsiary MM , Haydary A , et al. A common elements treatment approach for adult mental health problems in low‐ and middle‐income countries. Cognit Behav Pract. 2014;21(2):111–123. 25620867 10.1016/j.cbpra.2013.06.005 PMC4304666

[35] Craig SL , Eaton AD , Leung VW , Iacono G , Pang N , Dillon F , et al. Efficacy of affirmative cognitive behavioural group therapy for sexual and gender minority adolescents and young adults in community settings in Ontario, Canada. BMC Psychol. 2021;9(1):94. 34099063 10.1186/s40359-021-00595-6 PMC8183324

[36] Ye Z , Yu NX , Zhu W , Chen L , Lin D . A randomized controlled trial to enhance coping and posttraumatic growth and decrease posttraumatic stress disorder in HIV‐infected men who have sex with men in Beijing, China. AIDS Care. 2018;30(6):793–801. 29254367 10.1080/09540121.2017.1417534

[37] Bogart LM , Dale SK , Daffin GK , Patel KN , Klein DJ , Mayer KH , et al. Pilot intervention for discrimination‐related coping among HIV‐positive Black sexual minority men. Cult Divers Ethn Minor Psychol. 2018;24(4):541. 10.1037/cdp0000205 PMC6188818 29902020

[38] Craig SL , Austin A . The AFFIRM open pilot feasibility study: a brief affirmative cognitive behavioral coping skills group intervention for sexual and gender minority youth. Child Youth Serv Rev. 2016;64:136–144.

[39] Kidd R , Clay S , Chiiya C . Understanding and challenging HIV stigma: toolkit for action. Trainers Guide. The Change Project and ICRW. 2003.

[40] Lippman SA , Pettifor A , Rebombo D , Julien A , Wagner RG , Kang Dufour M‐S , et al. Evaluation of the Tsima community mobilization intervention to improve engagement in HIV testing and care in South Africa: study protocol for a cluster randomized trial. Implement Sci. 2017;12:1–13. 28095904 10.1186/s13012-016-0541-0 PMC5240325

[41] Lyons CE , Ketende S , Diouf D , Drame FM , Liestman B , Coly K , et al. Potential impact of integrated stigma mitigation interventions in improving HIV/AIDS service delivery and uptake for key populations in Senegal. J Acquir Immune Defic Syndr. 2017;74(Suppl 1):S52. 27930612 10.1097/QAI.0000000000001209 PMC5147043

[42] Rendina HJ , Cain DN , López‐Matos J , Ray M , Gurung S , Parsons JT . Measuring experiences of minority stress for transgender women: adaptation and evaluation of internalized and anticipated transgender stigma scales. Transgend Health. 2020;5(1):42–49. 32322687 10.1089/trgh.2019.0059 PMC7173692

[43] Ross MW , Smolenski DJ , Kajubi P , Mandel JS , McFarland W , Raymond FH . Measurement of internalized homonegativity in gay and bisexual men in Uganda: cross‐cultural properties of the Internalized Homonegativity scale. Psychol Health Med. 2010;15(2):159–165. 20391233 10.1080/13548500903527746

[44] Kalichman SC , Simbayi LC , Cloete A , Mthembu PP , Mkhonta RN , Ginindza T . Measuring AIDS stigmas in people living with HIV/AIDS: the Internalized AIDS‐Related Stigma Scale. AIDS Care. 2009;21(1):87–93. 19085224 10.1080/09540120802032627

[45] Kalichman SC , Shkembi B , Eaton LA . Finding the right angle: a geometric approach to measuring intersectional HIV stigma. AIDS Behav. 2022;26(Suppl 1):27–38. 34424389 10.1007/s10461-021-03437-z

[46] Kroenke K , Strine TW , Spitzer RL , Williams JB , Berry JT , Mokdad AH . The PHQ‐8 as a measure of current depression in the general population. J Affect Disord. 2009;114(1–3):163–173. 18752852 10.1016/j.jad.2008.06.026

[47] Spitzer RL , Kroenke K , Williams JB , Löwe B . A brief measure for assessing generalized anxiety disorder: the GAD‐7. Arch Intern Med. 2006;166(10):1092–1097. 16717171 10.1001/archinte.166.10.1092

[48] Sinclair VG , Wallston KA . The development and psychometric evaluation of the Brief Resilient Coping Scale. Assessment. 2004;11(1):94–101. 14994958 10.1177/1073191103258144

[49] Chesney MA , Ickovics J , Chambers D , Gifford A , Neidig J , Zwickl B , et al. Self‐reported adherence to antiretroviral medications among participants in HIV clinical trials: the AACTG adherence instruments. AIDS Care. 2000;12(3):255–266. 10928201 10.1080/09540120050042891

[50] Mayer KH , Stone VE . Strategies for optimizing adherence to highly active antiretroviral therapy: lessons from research and clinical practice. Clin Infect Dis. 2001;33(6):865–872. 11512092 10.1086/322698

[51] Johnson MO , Neilands TB , Dilworth SE , Morin SF , Remien RH , Chesney MA . The role of self‐efficacy in HIV treatment adherence: validation of the HIV Treatment Adherence Self‐Efficacy Scale (HIV‐ASES). J Behav Med. 2007;30:359–370. 17588200 10.1007/s10865-007-9118-3 PMC2423379

[52] Crabtree BF , Miller WL . Doing qualitative research. Sage Publications; 2023.

[53] Turan B , Budhwani H , Fazeli PL , Browning WR , Raper JL , Mugavero MJ , et al. How does stigma affect people living with HIV? The mediating roles of internalized and anticipated HIV stigma in the effects of perceived community stigma on health and psychosocial outcomes. AIDS Behav. 2017;21:283–291. 27272742 10.1007/s10461-016-1451-5 PMC5143223

[54] Taggart T , Jonathon Rendina H , Boone CA , Burns P , Carter J , English D , et al. Stigmatizing spaces and places as axes of intersectional stigma among sexual minority men in HIV prevention research. American Public Health Association; 2022. 10.2105/AJPH.2021.306676 PMC9241459 35763746

[55] Layland EK , Carter JA , Perry NS , Cienfuegos‐Szalay J , Nelson KM , Bonner CP , et al. A systematic review of stigma in sexual and gender minority health interventions. Transl Behav Med. 2020;10(5):1200–1210. 33044540 10.1093/tbm/ibz200 PMC7549413

[56] Andersen LS , Magidson JF , O'Cleirigh C , Remmert JE , Kagee A , Leaver M , et al. A pilot study of a nurse‐delivered cognitive behavioral therapy intervention (Ziphamandla) for adherence and depression in HIV in South Africa. J Health Psychol. 2018;23(6):776–787. 27121977 10.1177/1359105316643375 PMC5081274

[57] Terjesen MD , Doyle KA , Wade RL . Global adaptation and practice of cognitive behavioral therapy: an introduction. In: Terjesen MD , Doyle KA , editors. Cognitive behavioral therapy in a global context. Springer; 2022. p. 1–7.

[58] Jones MU , Ramadhani HO , Adebajo S , Gaydos CA , Kokogho A , Baral SD , et al. Seizing opportunities for intervention: changing HIV‐related knowledge among men who have sex with men and transgender women attending trusted community centers in Nigeria. PLoS One. 2020;15(3):e0229533. 32119701 10.1371/journal.pone.0229533 PMC7051043

[59] UNAIDS . Terminology guidelines. 2015.

