# Supplementary material for: Reducing stigma and promoting HIV wellness/mental health of sexual and gender minorities: RCT results from a group‐based programme in Nigeria
Source: J Int AIDS Soc. 2024 Jun 5;27(6):e26256. doi: 10.1002/jia2.26256 (PMC11151009; doi:10.1002/jia2.26256)
Supplement: Supplementary file 1 — Supplemental File 1. Additional details regarding study methods. [file JIA2-27-e26256-s001.docx]

**Supplemental File 1. Additional details regarding study methods**

**Power analysis**

Power calculations, based on the outcome of internalized stigma experienced by SGMs (comparing pre-post mean score with range 0.0-3.0, between the immediate and delayed group), suggested a sample size of 240 would be sufficient to detect an 0.2 difference, assuming a mean starting score of 1.5 (most conservative) and standard deviation of 0.5.

**Randomization procedures**

After recruitment, from separate listings of MSM and TGW enrolled participants, an investigator used a random numbers table and assigned individuals from each list to an intervention group (either immediate or delayed intervention); there were separate groups for MSM or TGW (given different experiences and social networks), participants and a subsample of HIV positive persons was included in each group. Within ten days of completion of the baseline survey, research assistants called participants to inform them of their randomization assignment.

**Qualitative procedures**

At baseline, survey participants were asked if they would be willing to be contacted for the future IDIs; over 90% agreed to be contacted. Participants were stratified by MSM/TGW status and by immediate/delayed intervention group status, then randomly selected for the IDIs. All who were contacted for the IDIs agreed to be interviewed.

Semi-structured IDI and FGD guides were developed jointly by a team of local and international researchers and implementers with expertise in developing and evaluating behavioural group interventions for SGM populations in Nigeria.

IDIs and FGDs were conducted in private rooms at the intervention implementation partner’s office by interviewers trained in qualitative interviewing with MSM and TGW; IDIs with program managers were conducted by phone. Interviews lasted approximately one hour and were audio-recorded This study adheres to standards for reporting qualitative research per the COREQ checklist.

**Qualitative data analysis**

An initial codebook was developed based on a priori codes informed by the research questions, IDI/FGD guides, and the literature. The codebook was iteratively revised by adding new codes that reflected emergent codes based on review of transcripts by two coders and a program manager. Half of the transcripts were double-coded; regular team meetings were held to resolve disagreement in coding among coders. Code reports and summaries for themes were then generated by study team members.

**Ethical approval**

All participants completed written informed consent for every study activity, with program participants completing one consent form for survey data collection, the intervention, and permission to be called for follow up in-depth interviews.

**References**

Tong A, Sainsbury P, Craig J. Consolidated criteria for reporting qualitative research (COREQ): a 32-item checklist for interviews and focus groups. Int J Qual Health Care. 2007;19(6):349–357. doi: 10.1093/intqhc/mzm042.
