# Supplementary material for: Reducing stigma and promoting HIV wellness/mental health of sexual and gender minorities: RCT results from a group‐based programme in Nigeria
Source: J Int AIDS Soc. 2024 Jun 5;27(6):e26256. doi: 10.1002/jia2.26256 (PMC11151009; doi:10.1002/jia2.26256)
Supplement: Supplementary file 2 — Appendix Table S1. Delayed group changes over time. [file JIA2-27-e26256-s002.docx]

**Appendix Table S1. Delayed group changes over time**

|  | **n** | **Effect of T1b (comparing to T0)** | **p-value** | **Effect of T1b (comparing to Round T1a)** | **p-value** |
| --- | --- | --- | --- | --- | --- |
| **IS-SGM** | 123 | -0.069 | 0.139 | 0.175 (unanticipated direction) | 0.000 |
| **IS-HIV** | 74 | -0.129 | 0.029 | 0.219  (unanticipated direction) | 0.003 |
| **Intersectional IS** | 74 | -0.072 | 0.196 | 0.214  (unanticipated direction) | 0.001 |
| **Depression** | 123 | -0.505 | 0.000 | -0.103 | 0.004 |
| **Anxiety** | 123 | -0.521 | 0.000 | -0.142 | 0.001 |
| **Coping** | 123 | 0.129 | 0.012 | -0.081 | 0.159 |

IS=internalized stigma; SGM=sexual and/or gender minority. *Controls (all measured at baseline):* age, education, how recruited, interviewer, man who has sex with men (MSM)/transgender woman (TGW) per intervention participation, relationship status, ability to meet basic needs, employment status (as well as HIV status, for models not limited to people living with HIV)
